# Structural insights into the translational infidelity mechanism

**DOI:** 10.1038/ncomms8251

**Published:** 2015-06-03

**Authors:** Alexey Rozov, Natalia Demeshkina, Eric Westhof, Marat Yusupov, Gulnara Yusupova

**Affiliations:** 1 Biologie Structurale Intégrative, Institut de Génétique et de Biologie Moléculaire et Cellulaire, Université de Strasbourg; CNRS, UMR7104; INSERM, U964, Illkirch 67400, France; 2 Architecture et Réactivité de l'ARN, Université de Strasbourg, Institut de Biologie Moléculaire et Cellulaire, CNRS, UPR9002, Strasbourg 67084, France

## Abstract

The decoding of mRNA on the ribosome is the least accurate process during genetic information transfer. Here we propose a unified decoding mechanism based on 11 high-resolution X-ray structures of the 70S ribosome that explains the occurrence of missense errors during translation. We determined ribosome structures in rare states where incorrect tRNAs were incorporated into the peptidyl-tRNA-binding site. These structures show that in the codon–anticodon duplex, a G·U mismatch adopts the Watson–Crick geometry, indicating a shift in the tautomeric equilibrium or ionization of the nucleobase. Additional structures with mismatches in the 70S decoding centre show that the binding of any tRNA induces identical rearrangements in the centre, which favours either isosteric or close to the Watson–Crick geometry codon–anticodon pairs. Overall, the results suggest that a mismatch escapes discrimination by preserving the shape of a Watson–Crick pair and indicate that geometric selection via tautomerism or ionization dominates the translational infidelity mechanism.

## Abstract (web summary)

Translation of mRNA into proteins is the least accurate process during genetic information transfer. Here the authors suggest—based on 11 high-resolution ribosome crystal structures—that the origin of protein missense errors involves molecular mimicry via tautomerism or ionization.

## Graphical abstract

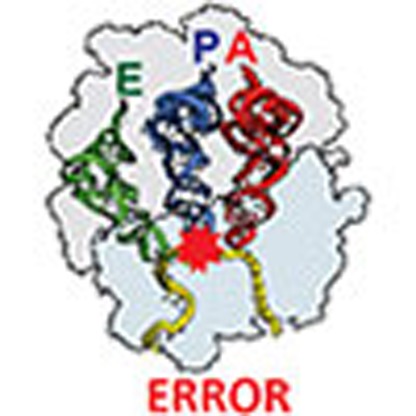

The misincorporation of amino acids into a polypeptide chain caused by incorrect decoding accounts for most missense errors during translation^1^,^2^. A comprehensive estimation of missense errors has always been a formidable experimental challenge due to the difficulties in detecting errors that constitute a small background in comparison with the abundance of correctly synthesized proteins. Today, the average efficiency of miscoding is estimated to be as high as 10^−3^–10^−4^ per amino-acid site^1^,^3^,^4^. Under normal physiological conditions, 18% of the proteins expressed from an average 400-codon-long gene contain at least one misincorporated amino acid^5^. More often, misincorporation is not deleterious and is important for the selective pressure on coding sequence evolution and cell fitness^5^; nevertheless, 10–50% of random substitutions affect protein function^1^,^6^. In bacteria and higher organisms, the rate of missense errors is similar, reflecting the universality of the genetic code.

In recent decades, X-ray crystallography has remained indispensable for understanding the molecular mechanisms of biological processes. Here we present several high-resolution structures of *Thermus thermophilus* 70S ribosomes programmed by templates carrying missense errors. The collection of our structures puts forward a decoding mechanism that, for the first time, sets the molecular basis behind the phenomenon of translational infidelity and is in good agreement with *in vivo* studies of the missense errors that occur during protein synthesis^4^,^7^,^8^.

## Results

### Mismatches in the peptidyl-tRNA-binding site

**Figure 1:**
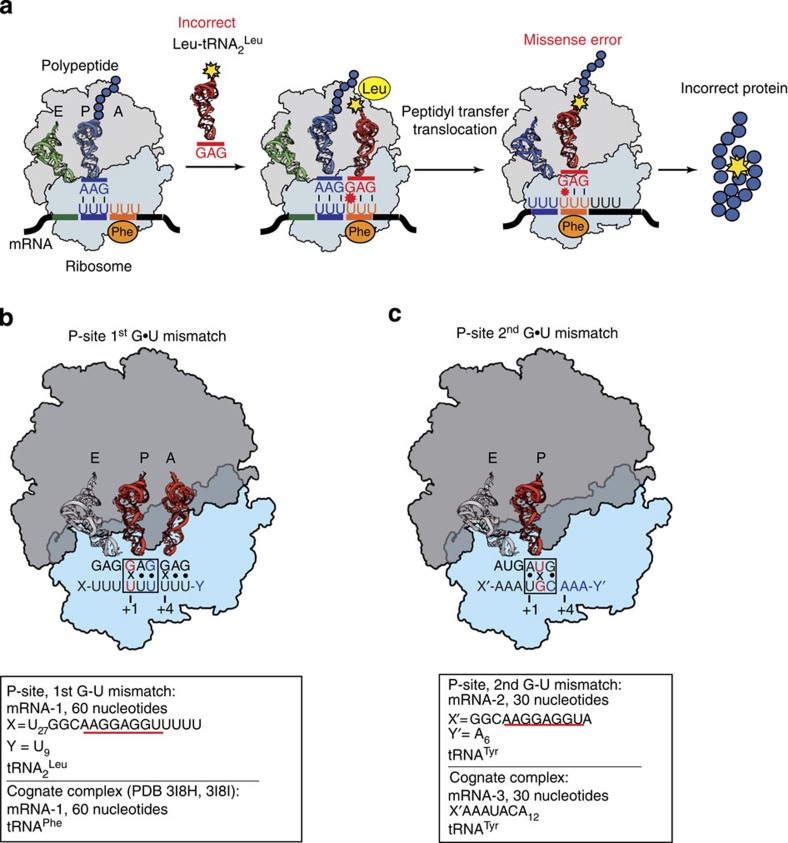
Miscoding of mRNA during protein synthesis. (**a**) The scheme depicts an event when the ribosome miscodes the phenylalanine codon by leucyl-tRNA^Leu^, which becomes translocated to the P-site and introduces an erroneous amino acid to a polypeptide chain. A, P and E define aminoacyl, peptidyl and exit tRNA-binding sites, respectively (**b**,**c**) Schematic representations of the 70S ribosome complexes where the G·U mismatch was modelled at the first (**b**) or second (**c**) position of the codon–anticodon duplex (framed). For each complex tRNA, which was used for the complex formation is specified together with the sequence of mRNA; in mRNA-1 and mRNA-2 the Shine–Dalgarno sequence is underlined. Cognate complexes used for comparisons are indicated below each schematic representation. The first nucleotides of the mRNA codons bound in the P- and A-sites are numbered (+1) and (+4), respectively.

**Figure 2:**
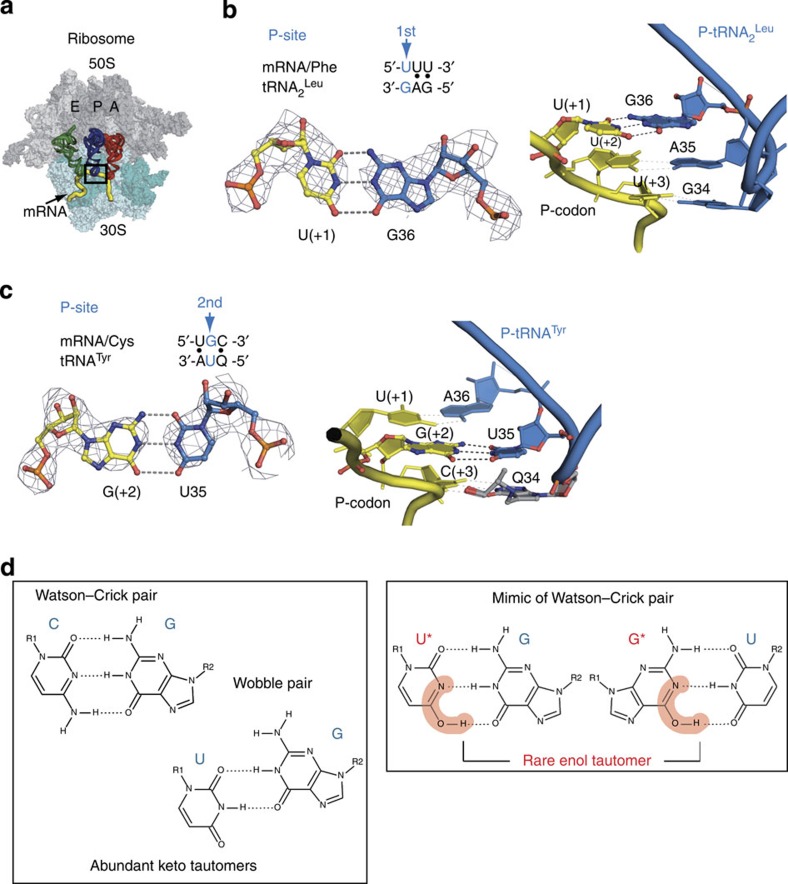
The G·U mismatch forms Watson–Crick-like pairs at the first and second codon–anticodon positions in the P-site of the 70S ribosome. (**a**) Region of codon–anticodon interactions in the P-site (framed) in the context of the full 70S ribosome; tRNA bound in the A-, P- and E-sites are represented by red, blue and green, respectively. (**b**,**c**) The G·U mismatch mimics Watson–Crick pair at the first (**b**; left) and second (**c**; left) positions of the codon–anticodon duplex (**b**,**c**; right). Putative hydrogen bonds formed by an anticipated tautomeric form of G or U have 2.8–3.2 Å length (see text). (**d**) Geometry of canonical Watson–Crick pair and non-canonical wobble pair formed by keto isomers (left panel). Watson–Crick-like pairs formed by rare enol tautomers of uracil or guanosine (right panel; marked by asterisk). In **b** and **d**, the following figures schemes of the codon–anticodon duplexes are indicated and arrows mark the described mismatches. All graphical representations were rendered by PyMol. For all figures, the density maps are contoured at 1.6−1.8 *σ* level.

We have successfully solved the structure of the 70S ribosome in two post-incorporation states (Fig. 1). In one case, we modelled the post-incorporation state based on the well-known *in vitro* miscoding system where polyuridylic acid served as a template and the leucyl-tRNA_2_^Leu^ served as a substrate for polyleucine synthesis (Fig. 1a,b)^9^,^10^. In this complex, the GAG anticodon of tRNA_2_^Leu^ formed two simultaneous G·U mismatches with the first and third positions of the phenylalanine UUU codon in the peptidyl-tRNA-binding site (P-site; Fig. 2a,b). Another messenger RNA (mRNA) construct and tRNA^Tyr^ let us model the second G·U mismatch with the cysteine codon UGC and the anticodon QUA bound in the P-site (Figs 1c and 2c). The structures of both states (Supplementary Table 1) revealed the remarkable finding that a G·U mismatch mimics a canonical Watson–Crick pair at either of the first two positions of the codon–anticodon duplex (Fig. 2b,c). Further analysis showed that the codon–anticodon duplexes containing G·U mismatches have an overall geometry that is identical to that of the corresponding cognate duplexes consisting of standard Watson–Crick pairs (Supplementary Fig. 1a)^11^. Moreover, we did not find any changes in the ribosomal environment (that is, A790, G926 and C1400 of the 16S ribosomal RNA (rRNA); ref. 12) of the near-cognate duplexes in the P-site. These results are particularly striking because in contrast to the restrictive decoding centre, where a G·U mismatch adopts the Watson–Crick geometry because of conserved ribosomal elements^13^, the P-site does not impose any obvious constraints on the codon–anticodon duplex that are discernible at 3 Å resolution. Nevertheless, ribosomal parts tightly hold the P-site transfer RNA^12^,^14^ with 16S residues forming A-minor groove-like contacts with two base pairs of the anticodon stem and with residue 790 blocking the anticodon stem on the other side (Supplementary Fig. 1b). In addition, C1400 in 16S rRNA stacks over the base pair at the third codon–anticodon position and G966 forms van der Waals contacts with the ribose of the 34th tRNA nucleotide (Supplementary Fig. 1b). At the same time, the mRNA path is also constrained by the ribosome by a bend (the E/P-kink)^11^,^14^ at the phosphate between the last (−1) and the first (+1) nucleotides that is stabilized by hydrogen bonds. Moreover, the P-codon is fixed by several interactions of the ribosome with its sugar-phosphate backbone (Supplementary Fig. 1c,d).

### Mismatches in the aminoacyl-tRNA-binding site

To expand our previous findings^13^, we investigated non-Watson–Crick pairs other than the G·U mismatch in the 70S ribosomal decoding centre. For this study, we chose a ‘challenging' pyrimidine–purine mismatch, C·A, and a purine–purine mismatch, A·A, and we solved seven high-resolution structures where these mispairs were placed at either of the first two positions of a codon–anticodon duplex in the aminoacyl-tRNA-binding site (A-site) (Supplementary Table 1). We also solved a control structure to demonstrate that the decoding centre is specific in our system and can only bind cognate tRNA or near-cognate tRNA that resembles cognate substrates. In this control complex, where mRNA programmed the ribosome with the AAA codon in the A-site and where tRNA^Phe^ with the GAG anticodon was given as a substrate, no binding of tRNA^Phe^ to the A-site was detected (Supplementary Table 1).

**Figure 3:**
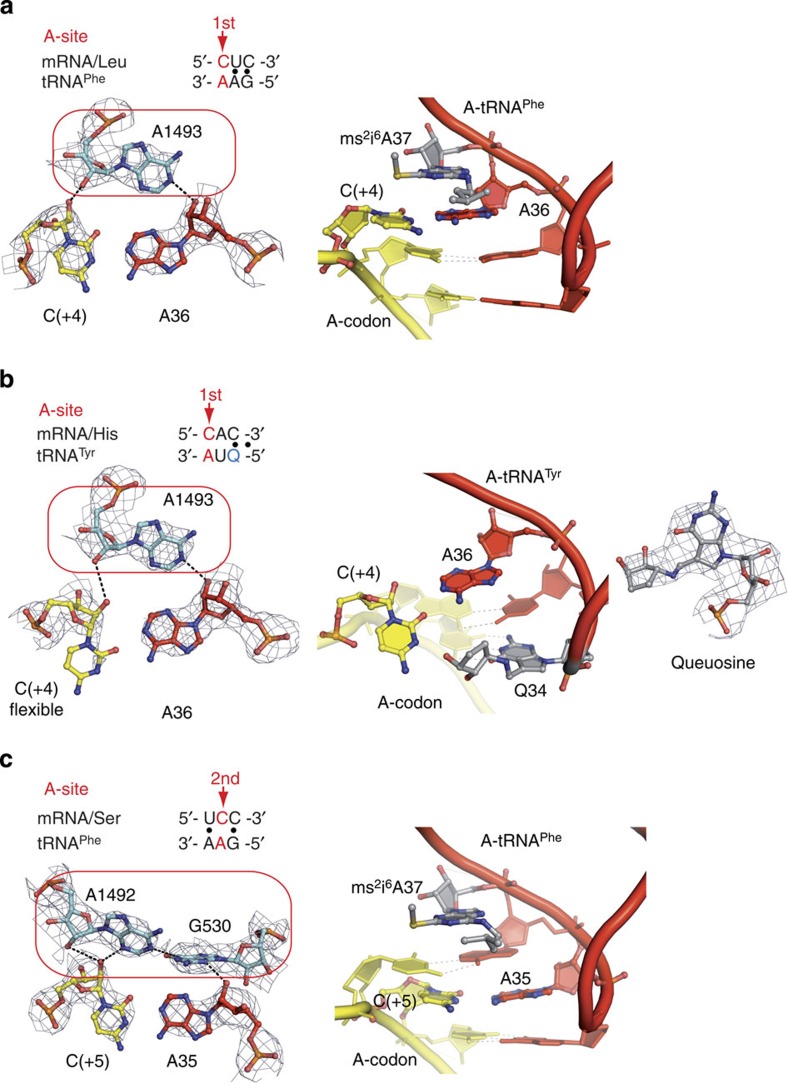
The C·A mismatch does not form a stable pair in the 70S ribosome-decoding centre. (**a**,**b**) The C·A mismatch at the first position of the codon–anticodon duplex in the absence (**a**) or presence (**b**) of the queuosine modification in the tRNA anticodon. In **a**, the left panel shows that A1493 in 16S rRNA prevents strong pairing interactions in the C·A mismatch by constraining its sugar-phosphate backbone by hydrogen bonding. In **b**, the left and middle panels demonstrate a lack of pairing in the C·A mismatch reflected by the weak electron density signal corresponding to the cytosine base and misshaping of the mini-helical structure due to displacement of the cytosine by queuosine (right). (**c**) The C·A mismatch at the second position of the codon–anticodon duplex; as in **a** and **b**, the left panel depicts conserved A1492 and G530 in 16S rRNA tightening around the mismatch and contorting it. The density maps are contoured at 1.8 σ level. In **a**–**c**, the right panels depict overall geometry of the mismatches in the codon–anticodon mini-helices.

**Figure 4:**
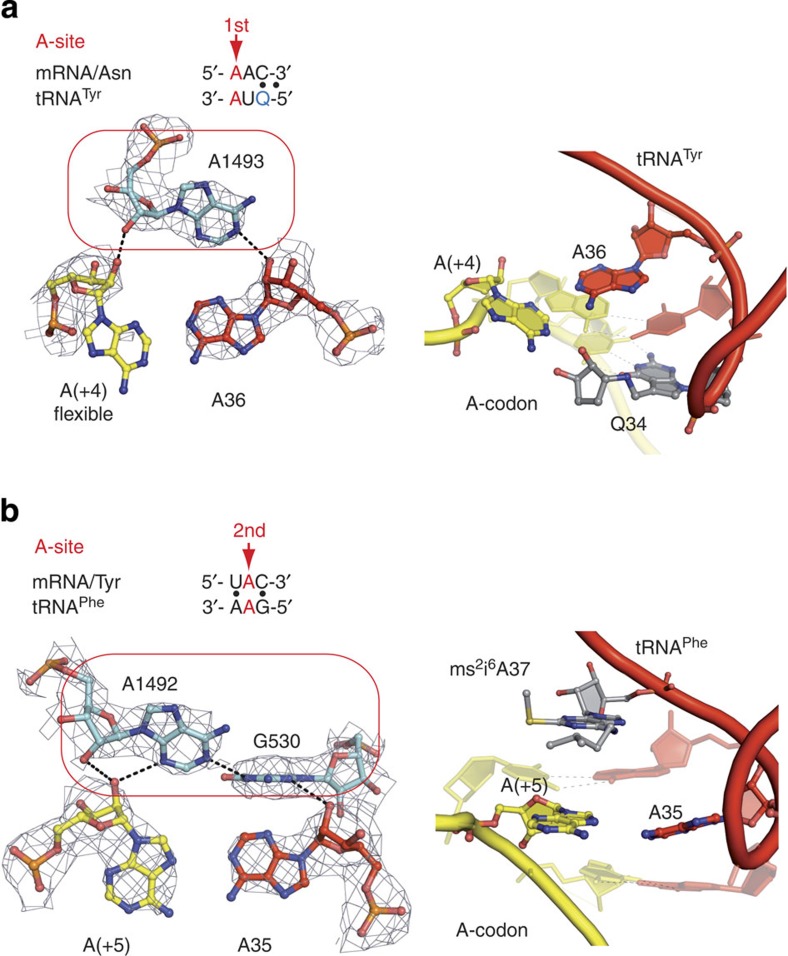
The A·A mismatch in the decoding centre of the 70S ribosome. The left panels demonstrate the A·A mismatch at the first (**a**) and second (**b**) positions of the codon–anticodon duplexes. As for the C·A mismatches, A1493 (**a**) and A1492 with G530 (**b**) in 16S rRNA constrain the sugar-phosphate backbones of the first and second mispairs by hydrogen bonding. In **a**, the exact position of the codon adenosine was not detectable and the figure shows one of the possible positions of this nucleobase; in **b**, the distances between adenosines exceed 3.6 Å demonstrating absence of strong interactions. In **a** and **b**, the right panels depict overall geometry of the mismatches in the codon–anticodon mini-helices.

The analysis of the models reinforced one of our earlier significant findings that the binding of near-cognate and cognate tRNA to the 70S ribosome induces identical rearrangements of (i) the small ribosomal subunit (that is, shift of the shoulder domain by 2–3 Å) and (ii) the decoding centre itself (Figs 3 and 4; see Supplementary Fig. 2c and Supplementary Movie)^13^. Independently of which near-cognate tRNA was present in the centre, the conserved A1493 and A1492/G530 of the 16S rRNA stabilized the first and second codon–anticodon pairs in a manner identical to that of the cognate models (Figs 3 and 4). In addition, the conserved A1913 of helix 69 (H69) in the 23S rRNA stabilized the first codon–anticodon position through contacts with the 37th nucleotide of the near-cognate and cognate tRNA anticodon loops^14^,^15^.

A close-up analysis at every mismatch revealed that despite the stabilization of the sugar-phosphate backbones by A-minor groove interactions with the A1492/1493 and G530 of the 16S rRNA, the nitrogen bases of the A·A and C·A mispairs did not interact stably (Figs 3 and 4; see Supplementary Fig. 3). Thus, the C·A mismatch at the first codon–anticodon position was shifted from the Watson–Crick geometry; however, the shift did not quite reach the wobble position, possibly reflecting a metastable or average state (Fig. 3a). The resulting interatomic distances and putative bond angles of the mismatch suggested that the formation of hydrogen bonds was highly unlikely. When the first C·A mismatch was modelled with tRNA^Tyr^, the presence of a queuosine modification^16^, which was not visible in previous structures of tRNA^Tyr^ 13, at the first anticodon position led to the displacement of the cytosine from the codon–anticodon helix (Fig. 3b) and distortion of the latter. This change emphasized the lack of stable interactions in the C·A mispair and pointed to an amending role of tRNA modifications in translational accuracy. The base pair geometry of the C·A at the second codon–anticodon position was very similar to that of the first C·A mismatch in the absence of queuosine (Fig. 3c).

No definite density signal was observed for the base of the mRNA adenosine in the structure with the first A·A mispair in the codon–anticodon duplex, demonstrating its mobility (Fig. 4a). One of the possible conformations could be stabilized by the queuosine of tRNA^Tyr^ (as was the case for the first cytosine (Fig. 3b); however, any interaction with the anticodon adenosine was unlikely. The structure with the A·A mismatch at the second codon–anticodon position provided further evidence of the canonical constraints of the 70S decoding centre. Limited by A1492/G530 and stacking interactions with the standard Watson–Crick pairs at the first and third duplex positions, the adenosine of mRNA was found in the *syn* conformation with its Hoogsteen plane exposed to the Watson–Crick surface of the opposing adenosine in tRNA (Fig. 4b). Nevertheless, interatomic distances of >3.6 Å excluded possibility of strong interactions between the two adenosines, stressing the fact that the 70S decoding centre suppresses the formation of non-Watson–Crick pairs by restrictive steric and geometrical constraints.

We also determined structures with the A·A and C·A mismatches at the first and second positions of the codon–anticodon duplexes, respectively, in the presence of the miscoding aminoglycoside paromomycin. Binding of the antibiotic did not affect the geometry of the mismatches and resulted in the same relaxation of the decoding pocket and shift of H69 towards tRNA that we described previously (Supplementary Fig. 4)^13^.

## Discussion

The results obtained for the G·U mismatches presented here as well as those that were previously published^13^ are closely related to the work of Topal and Fresco, who discussed base-pairing schemes and attempted to explain translational errors^17^. Their work extended the hypothesis of Watson and Crick, who suggested that spontaneous mutagenesis in replication is caused by a base adopting one of its rare tautomeric forms^18^. Topal and Fresco implied that a non-Watson–Crick pair matching the dimensions of a canonical Watson–Crick pair should be accepted and expressed by the ribosome. They also postulated that the internal ribosome environment influences the keto–enol tautomeric equilibrium of mRNA and tRNA by locking the isomeric state after binding to the ribosome. In some cases, it leads to the rare enol tautomers being favoured over the more abundant keto isomers^17^ (Fig. 2d).

Although the 3 Å resolution of our models is not sufficient to distinguish between the two tautomeric forms, the observed Watson–Crick-like geometries for the G·U pairs can be rationalized by the presence of enol tautomers in the P-site (Fig. 2d). Most likely the formation of minor tautomers of G or U either in mRNA or in tRNA happens before their binding to the ribosome, that is, in solution. While this paper was under review, NMR relaxation dispersion measurements showed that in RNA duplexes a wobble G·U pair exists in dynamic equilibrium with short-lived, low-populated Watson–Crick-like pairs that are stabilized by rare enolic or anionic bases (see page 318 in ref. 19). Our present structure with the P-site G·U mismatches trapped in a Watson–Crick geometry, as well as the previous report^13^ on the A-site with G·U in Watson–Crick-like geometries, fully support these observations. According to the NMR relaxation dispersion calculations, frequencies of occurrence of minor enolic or anionic bases spans the range of 10^−3^–10^−5^ that are not far from the accepted translation error rate of 10^−3^–10^−4^ (refs 3, 4).

In an analogous fashion to the mRNA P/A-kink^13^,^14^,^20^, the mRNA constraints between the E-codon and P-codon^11^,^14^ together with the tight ribosome grip surrounding the tRNA anticodon stem-loop^12^,^14^ contribute to the fixing of the P-site codon–anticodon mini-helix in place (Supplementary Fig. 2b–d). Thus, this fixation would restrain the first codon nucleotide from the shift necessary to form a wobble pair. For the second codon–anticodon position, a bend caused by a wobble G·U pair would be also forbidden because the tight shape of the codon–anticodon mini-helix is defined by the tRNA anticodon rigid structure and a tertiary structure of tRNA stabilized by the ribosome^12^,^14^.

However, the above scheme with the assumption of an equal occurrence of tautomers or anionic bases at all the three codon or anticodon positions does not explain the presence of a G34·U3 pair in a standard wobble geometry at the third position of the codon–anticodon duplex (Supplementary Fig. 2a). It seems likely that, compared with the two other anticodon positions fixed by stacking interactions, the tRNA nucleotide 34 that forms the third pair exhibits some extent of freedom due to its apical location in the U-turn fold of an anticodon loop^21^ (Supplementary Fig. 2b). Its chemical state can also be dictated by the composition of the loop, including modifications^22^,^23^.

The present results suggest that the extent of molecular adaptability allowing a non-complementary pair to form an isosteric pair with the Watson–Crick-like geometry defines the probability and efficiency of a miscoding event. We can infer that among the described complexes, those ribosomes bearing mRNA and tRNA with G·U mismatches would be by far the most stable^24^, while those bearing C·A and A·A mismatches would be less homogeneous and less stable. In the context of the kinetic scheme of decoding^3^, such complexes will be prone to dissociation rather than translocation. Recently published studies dealing with the *in vivo* frequencies of mismatches in translation ranked G·U, U·U or C·U mismatches as the most frequent, and A·A and C·A mispairs as the least probable errors during protein synthesis^4^,^8^, fully in accordance with the conclusions derived from our structural data.

**Figure 5:**
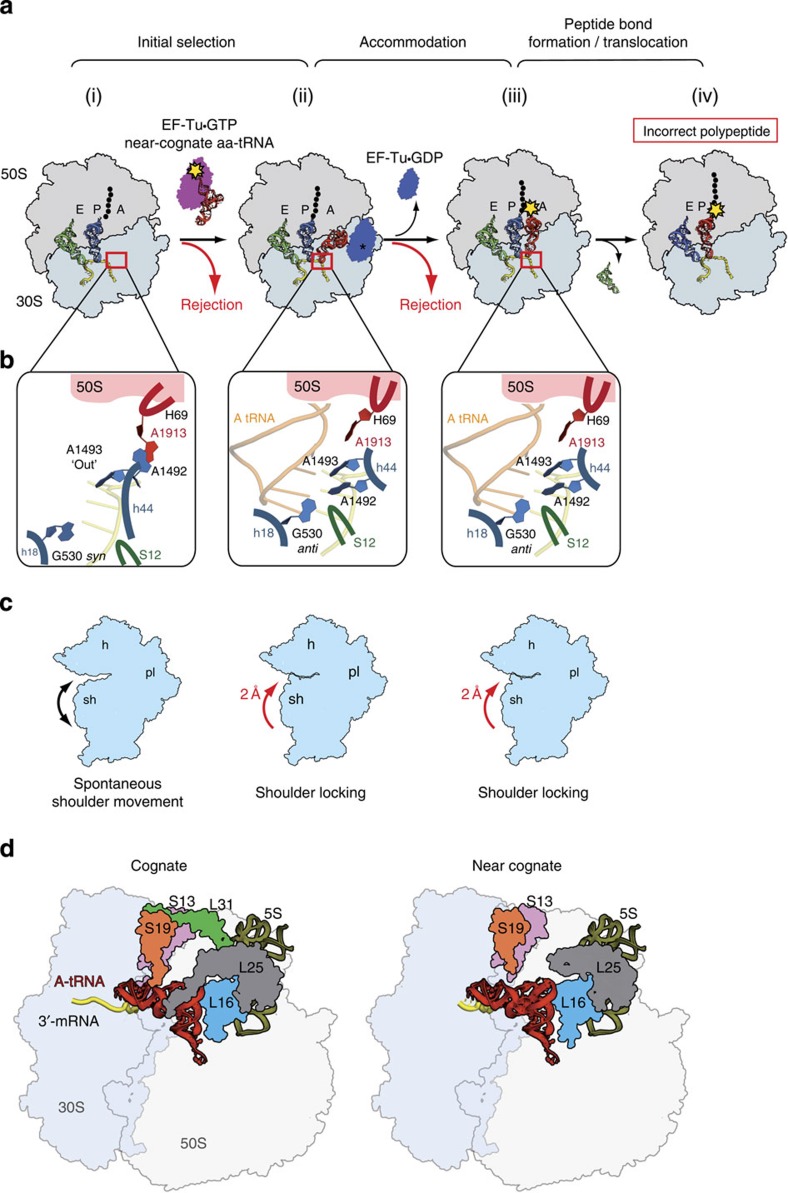
Proposed mechanism of translational infidelity. (**a**) Misincorporation of an amino acid by the ribosome. The main steps of the tRNA selection process are shown including hydrolysis of GTP (black asterisk) by elongation factor Tu on establishing codon–anticodon interactions in the decoding centre; (i–iv) indicate sequential steps of the process (see the text). (**b**) Conformation of the main nucleotides of the decoding centre without tRNA (left), bound by cognate or near-cognate aa-tRNA at the initial recognition step^45^ (middle) and at the final step of accommodation (right). The crucial nucleotides of 16S and 23S rRNA are shown in cyan and red, respectively. Ribosomal protein S12, which belongs to the shoulder domain of the small ribosomal subunit and additionally restricts the second codon–anticodon pair^46^, is depicted in green. The three nucleotides of the mRNA codon in the A-site are numbered according to the standard system (see legend to Fig. 2). (**c**) Overall states of the small ribosomal subunit during selection of tRNA. The left panel pictures spontaneous movement of the shoulder domain (black arrows) when the decoding centre is unoccupied; the middle and right panels show that the shoulder is shifted and stabilized on binding of near-cognate tRNA during the initial selection step and further accommodation (see text); sh, h, pl denote the shoulder, head and platform domains of the small ribosomal subunit, respectively. (**d**) Strengthening of cognate tRNA binding by protein tails from the small (S) and large (L) ribosomal subunits. Fastening of cognate tRNA in the A-site is also represented by formation of the additional intersubunit bridge between protein L31 and proteins S13 and S19 (PDB codes 3I8H and 3I8I). The protein extensions are disordered when near-cognate tRNA is bound in the A-site (present work; the model of the 70S ribosome with tRNA_2_^Leu^ bound to the UUU codons in the P- and A-sites). The absence of additional stabilization by the proteins can promote dissociation of near-cognate tRNA from the ribosome.

Taken together, the present structures along with our earlier models of the 70S ribosome primed by long templates and native tRNA^11^,^13^,^15^ provide an extensive library of various states of the P-site and the decoding centre on the 70S ribosome. Our models suggest an advanced mechanism of decoding that, for the first time, describes how a missense error can skip discrimination, leading to translational infidelity (Fig. 5). Although our structures were obtained in a non-enzymatic system, numerous lines of experimental data support the unified principles that underlie the basic functions of the ribosome and hence allow us to generalize the proposed mechanism.

For the present mechanism of decoding (Fig. 5), we want to emphasize the crucial role of the large ribosomal subunit and, in particular, its helix 69 that forms the intersubunit bridge B2a (ref. 25) and acts as a regulator of nucleotide A1492 of the 16S rRNA in the decoding centre^15^. The tRNA selection begins with the binding of tRNA to the unoccupied centre (Fig. 5a, i), which is predisposed to accept tRNA^26^. In this unoccupied centre, A1493 protrudes from helix 44 of the 16S rRNA and is ready to interact with the minor grove of the first pair of the codon–anticodon duplex. Furthermore, A1492 stacks over A1913 in H69 of the 23S rRNA^15^ (Fig. 5b, left). The kink of the mRNA between the P- and A-codons pre-positions the sugar phosphate of the first nucleotide so that it cannot be displaced towards the major groove of the codon–anticodon mini-helix^11^,^13^,^14^,^20^. Further tightening of the centre occurs independently of the cognate or near-cognate tRNA nature and can be described by two major rearrangements of the ribosome (Fig. 5a, ii). First, on tRNA binding, the anticodon loop contacts the tip of H69 in the 23S rRNA and apparently disrupts the A1492/A1913 stacking (Supplementary Movie). As a result, A1492 and A1913 undergo local rearrangements, resulting in the formation of minor groove interactions between A1492 and the second codon position (Fig. 5b, middle). These rearrangements essentially define the decoding pocket from the side of mRNA. The second rearrangement is the slight movement of the shoulder domain of the small subunit towards the anticodon loop that brings together G530 (which switches its conformation from *syn* to *anti*) with the second anticodon position and finalizes the formation of the decoding centre (Fig. 5b, middle). Considering early evidence of non-enzymatic polypeptide synthesis^27^,^28^, we suggest that the shoulder movement represents inherent ribosomal dynamics (Fig. 5c; Supplementary Movie) that underlies other essential functions of the ribosome, such as the translocation assisted by spontaneous ratcheting and swivelling of the small subunit^29^. Most likely, this inherent movement of the shoulder domain is locked on tRNA binding in a state that completes the formation of the discriminatory centre. However, the detailed kinetics of this rearrangement remains an open question.

A codon–anticodon duplex entrapped in the decoding centre is then tested for steric complementarity to the restrictive mould of the decoding centre. In this framework, cognate tRNA will be efficiently incorporated because of its ability to form stable Watson–Crick pairs with the first two codon positions. Most near-cognate tRNAs will be sorted off due to steric clashes within a codon–anticodon pair or with limiting constraints of the centre like in the case of a standard wobble G·U pair (Supplementary Fig. 5) and the large free-energy cost required to fit in the centre (Fig. 5a, i–ii). However, a few of the erroneous RNA molecules will escape discrimination because of their capability to form Watson–Crick-like interactions. During the final steps of selection, some of these near-cognate tRNA molecules can still dissociate from the ribosome due to the instability of the formed pairing interactions (Fig. 5a, ii–iii). As we proposed earlier^15^, the extensions of some ribosomal proteins can perform an additional ‘discriminatory' role against near-cognate tRNA at this step (Fig. 5d). In the steady-state enzymatic system, the falloff of tRNA would lead to non-productive hydrolysis of GTP by elongation factor Tu^30^, which catalyses the tRNA delivery to the ribosome and hydrolyses GTP after establishing the codon–anticodon interactions in the decoding centre (Fig. 5a). However, some near-cognate pairs, such as the G·U pair, will maintain Watson–Crick geometry via rare tautomeric or anionic forms and will be accommodated in the decoding centre and further translocated to the P-site, resulting in misincorporation of an amino acid into a polypeptide chain (Fig. 5a, iv). This scenario agrees with the studies of the tRNA selection process using the single-molecule fluorescence resonance energy transfer approach^31^,^32^ and fits well into the contemporary kinetic scheme of the process that suggests that decoding on the ribosome is evolutionally optimized towards a higher speed of translation at the cost of fidelity^3^.

Our data provide evidence that steric complementarity and shape acceptance but not the number of hydrogen bonds between the decoding centre and a codon–anticodon duplex play the discriminatory role during decoding^33^. Our translational infidelity mechanism finds support in recent studies where multiple 2′-fluoro substitutions in mRNA disrupting the hydrogen bonds between the mRNA codon and the decoding centre only had a modest effect on the tRNA selection efficiency^34^. Our models further reinforce the specific role of tautomerism or base ionization in infidelity mechanisms of other biological processes, such as DNA replication^35^,^36^, and we propose an original view of the phenomena that may involve non-canonical Watson–Crick pairs, for example, in non-canonical decoding^37^,^38^ or during the initiation from alternative start codons^39^,^40^.

## Methods

### Ribosome purification and complex formation

The 70S ribosomes from the *T. thermophilus* strain H8 were purified according to the following protocol.

The cells (100 g) were washed with 1 l of buffer A (150 mM MgCl_2_, 500 mM NH_4_Cl, 40 mM Tris-HCl pH 7.5, 1.5 mM EDTA-Na_2_, 1 mM DTT) and then resuspended in 100 ml of the same buffer. All the procedures were performed at 4 °C. DNase (1 unit per ml) together with phenylmethylsulphonyl fluoride (1 μg ml^−1^) were added and the cells were disrupted by the French Press (or microfluidizer). The debris was removed by 30 min centrifugation at 30,000*g* and the resultant supernatant (S30) was layered on the first cushion (1.5 M sucrose, 0.68 M CsCl, 150 mM MgCl_2_, 20 mM Tris-HCl pH 7.5, 1.5 mM EDTA-Na_2_, 1 mM DTT) and centrifuged at 100,000*g* for 20 h using the SW28 rotor (Beckman). The bottom 5-ml cushion fractions were collected and diluted three times with buffer B (50 mM MgCl_2_, 150 mM NH_4_Cl, 20 mM Tris-HCl pH 7.5, 0.5 mM EDTA-Na_2_, 1 mM DTT) and then layered on the second cushion (1.8 M sucrose, 0.8 M CsCl, 150 mM MgCl_2_, 20 mM Tris-HCl pH 7.5, 1.5 mM EDTA-Na_2_) and centrifuged at 100,000*g* for 40 h in the SW28 rotor (Beckman). The bottom 4-ml cushion fractions were collected and dialysed against buffer B. The 4 M solution of ammonium sulfate (NH_4_)_2_SO_4_ was added to the dialysed fractions to the final concentration of 1 M.

The ribosomes (500 mg) were loaded on a 200-ml column of Toyopearl Butyl 650S equilibrated in buffer C (10 mM MgCl_2_, 400 mM NaCl, 20 mM Tris-HCl pH 7.5, 0.5 mM EDTA-Na_2_, 1 mM DTT) containing 1 M (NH_4_)_2_SO_4_. The column was washed with two volumes of buffer C with 0.8 M (NH_4_)_2_SO_4_ and the ribosomes were then eluted by 900 ml of a reverse gradient of (NH_4_)_2_SO_4_ (from 80 to 40%) keeping other components of buffer C constant (the flow rate 6 ml min^−1^, the fraction volume 12 ml). The peak of 70S ribosomes was collected and concentrations of (NH_4_)_2_SO_4_ and MgCl_2_ were adjusted to 1 M and 50 mM, respectively. The 70S peak was then concentrated by step-wise elution from 200 ml Toyopearl Butyl 650S equilibrated in buffer C containing 1 M (NH_4_)_2_SO_4_. Finally, the ribosomes were dialysed against buffer D (10 mM Mg(CH_3_COO)_2_, 50 mM KCl, 10 mM NH_4_Cl, 1 mM DTT, 10 mM HEPES, pH 7.5), applied on the 5–20% sucrose gradient prepared in buffer D and further centrifuged at 15,400 r.p.m. for 17 h in the SW28 rotor (Beckman). The peaks corresponding to 70S ribosomes were combined and the ribosomes were pelleted by ultracentrifugation at 45,000 r.p.m. for overnight in the type 45 Ti rotor (Beckman). The 70S ribosome pellet was resuspended in buffer D with 5 mM HEPES, pH 7.5, flash frozen in liquid nitrogen and stored in small aliquots at –80 °C.

Uncharged native individual tRNA^Phe^, tRNA^Tyr^ and tRNA^fMet^ from *Escherichia coli* were purchased from Chemical Block (Russia). All mRNA constructs whose sequences are specified below were from Thermo Scientific (USA) and deprotected following the supplier procedure. Aminoglycoside antibiotic paromomycin was purchased from Sigma-Aldrich.

The ribosomal complexes were formed in 10 mM Tris-acetate, 40 mM KCl, 7.5 mM Mg(CH_3_COO)_2_, 0.5 mM DTT at pH 7.0 at 37 °C. For all complexes, the 70S ribosomes (3 μM) were incubated with fivefold stoichiometric excess of mRNA and three to fivefold excess of tRNA. For the complexes containing the G·U mismatches in the P-site, the 70S ribosomes (3 μM) were incubated with mRNA-1 and tRNA_2_^Leu^ or mRNA-2 and tRNA^Tyr^ (Fig. 1b,c) for 15 min. For comparison of the near-cognate complexes with G·U at the first codon–anticodon position, we used our previous model of the 70S ribosome with cognate tRNA^Phe^ bound to the UUU codon in the P-site^11^ (Fig. 1b). For the second G·U mismatch, we made a separate control complex by incubating 70S ribosomes with mRNA-3 and tRNA^Tyr^ (Fig. 1c).

For the near-cognate complexes with mismatches in the decoding centre, mRNA sequences were 27–30 nucleotides long and contained 5′-GGC.AAG.GAG.GCA.AAA-3′ (Z) at the 5′-end^20^. The exact sequences were as follows: mRNA-4=ZAUGCUCA_9_; mRNA-5=ZAUGCACA_9_; mRNA-6=ZAUGUCCA_9_; mRNA-7=ZAUGAACA_6_; and mRNA-8=ZAUGUACA_6_ (the start codon and the Shine–Dalgarno sequence are underlined).

The 70S ribosomes (3 μM) were pre-incubated with mRNA-4, mRNA-5, mRNA-6, mRNA-7 or mRNA-8 and tRNA^fMet^ for 15 min to fill the P-site. The complexes modelling the C·A mismatch at the first and second codon–anticodon positions were obtained by incubating tRNA^Phe^ with the 70S/tRNA^fMet^/mRNA-4 and 70S/tRNA^fMet^/mRNA-6 mixtures, respectively, for 30 min. The C·A mismatch at the first position was also prepared by incubation of the 70S/tRNA^fMet^/mRNA-5 mixture with tRNA^Tyr^. The complexes with the A·A mismatch at the first and second codon–anticodon positions were made by addition of tRNA^Tyr^ and tRNA^Phe^ to the 70S/tRNA^fMet^/mRNA-7 and 70S/tRNA^fMet^/mRNA-8 mixtures, respectively, and incubated as described above.

Complexes with paromomycin were obtained by including the antibiotic (60 μM) into the incubation mixture containing 70S/tRNA^fMet^/mRNA-6/tRNA^Phe^ and 70S/tRNA^fMet^/mRNA-7/tRNA^Tyr^.

Crystals were grown at 24 °C via vapour diffusion in sitting-drop plates (CrysChem, Hampton Research). The ribosomal complex (2 μl) containing 2.8 mM Deoxy Big Chaps (CalBioChem) was mixed with the equal volume of the crystallization solution (3.9–4.2% (w/v) PEG 20k, 3.9–4.2% (w/v) PEG550mme, 100 mM Tris-acetate, pH 7.0, 100 mM KSCN). The crystals grew for 2–3 weeks and were then dehydrated by exchanging the reservoir for 60% (v/v) 2-methyl-2,4-pentanediol. Before freezing in the nitrogen stream, crystals were then cryo protected by the addition of 30% (v/v) 2-methyl-2,4-pentanediol and 14 mM Mg(CH_3_COO)_2_.

### Data collection, processing and structure determination

Data for all complexes were collected at the PXI beamline of Swiss Light Source, Switzerland, at 100 K. A very low dose mode was used and huge redundancy data were collected^41^. The data were processed and scaled using XDS^42^. All crystals belong to space group P2_1_2_1_2_1_ and contain two ribosomes per asymmetric unit. One of the previously published structures^13^, with tRNA, mRNA and metal ions removed, was used for refinement with Phenix^43^. The initial model was placed within each data set by rigid body refinement with each biopolymer chain as a rigid body. This was followed by initial coordinate refinement. The resulting electron density maps were inspected in Coot^44^ and the tRNA and mRNA ligands were built in. During several cycles of manual rebuilding followed by coordinate and isotropic B-factor refinement, magnesium ions were added and the final refinement round took place. The data collection and refinement as well as model geometry statistics are presented in Supplementary Table 1.

## Additional information

**Accession codes:** The atomic coordinates and structure factors for the reported crystal structures have been deposited in the Protein Data Bank under accession codes 4WQ1 (A-site: 1st C·A with tRNA^Tyr^), 4WQR (A-site: 1st C·A with tRNA^Phe^), 4WR6 (A-site: 1st A·A), 4WRA (A-site: 1st A·A with paromomycin), 4WRO (A-site: 2nd C·A), 4WSD (A-site: 2nd C·A with paromomycin), 4WT1 (A-site: 2nd A·A), 4WSM (P-site: 1st G·U), 4WU1 (P-site: 2nd G·U), 4WZD (P-site: 2nd G·C) and 4WZO (A-site: control)

**How to cite this article:** Rozov, A. *et al*. Structural insights into the translational infidelity mechanism. *Nat. Commun*. 6:7251 doi: 10.1038/ncomms8251 (2015).

## Supplementary Material

Supplementary Figures and Table Supplementary Figures 1-5, Supplementary Table 1 and Supplementary Reference

Supplementary Movie 1 Conformational changes accompanying formation of the decoding center on the 70S ribosome
