# Supplementary material for: Structural insights into the translational infidelity mechanism
Source: Nat Commun. 2015 Jun 3;6:7251. doi: 10.1038/ncomms8251 (PMC4468848; doi:10.1038/ncomms8251)
Supplement: Supplementary Figures and Table — Supplementary Figures 1-5, Supplementary Table 1 and Supplementary Reference [file ncomms8251-s1.pdf]

Supplementary Information

Structural insights into the translational infidelity mechanism

Alexey Rozov[1,3], Natalia Demeshkina[1,3], Eric Westhof[2], Marat Yusupov[1]& Gulnara Yusupova[1]

[1]Biologie structurale intégrative, Institut de Génétique et de Biologie Moléculaire et Cellulaire, Université de Strasbourg; CNRS, UMR7104; INSERM, U964, Illkirch, 67400, France

[2]Architecture et Réactivité de l'ARN, Université de Strasbourg, Institut de Biologie Moléculaire et Cellulaire, CNRS, UPR 9002, Strasbourg, 67084, France

[3]These authors contributed equally to the work

Correspondence should be addressed to G.Y. (gula@igbmc.fr)

Supplementary Information

Supplementary Figures 1-5

Supplementary Table 1

Supplementary Movie

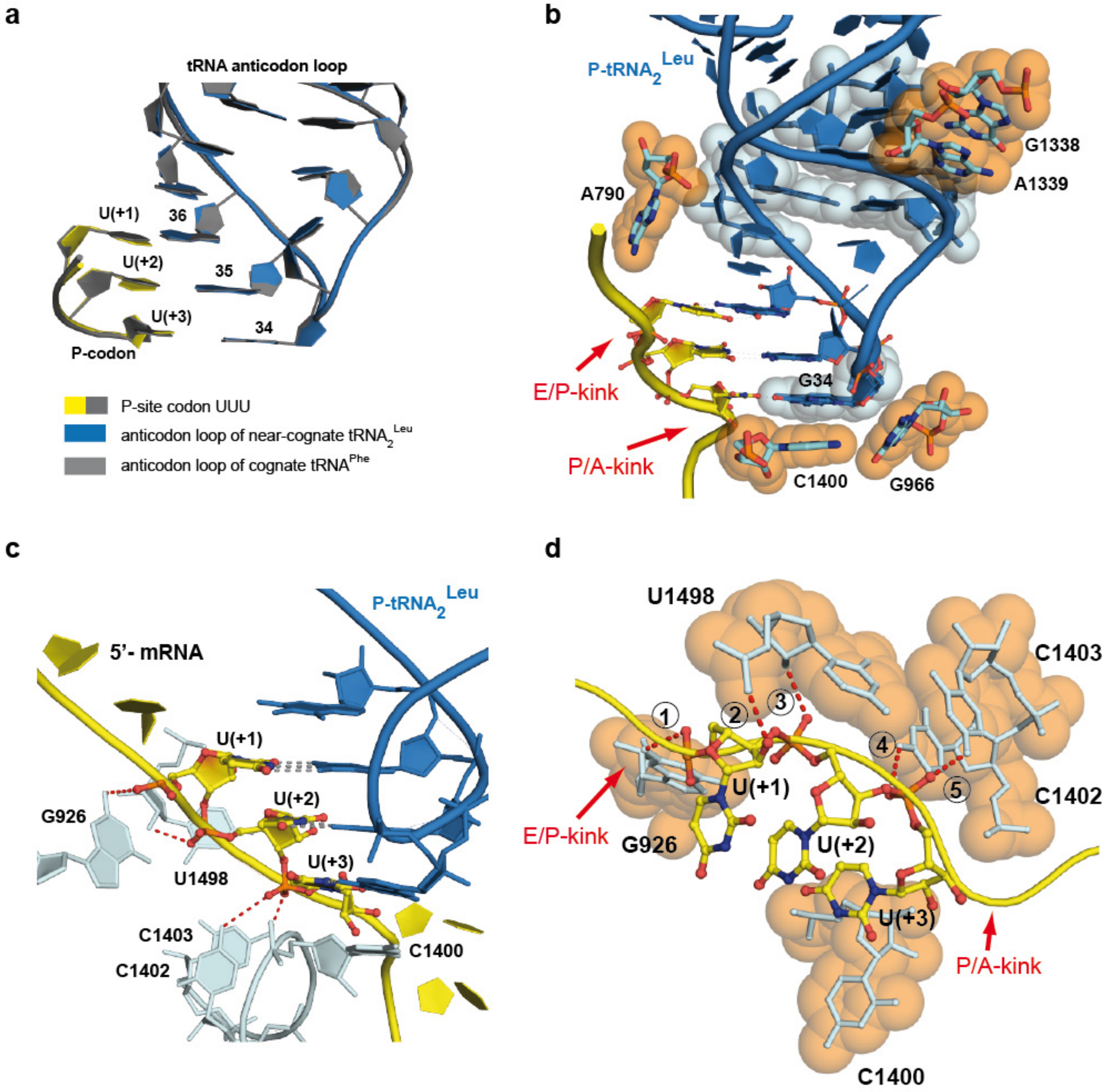

Supplementary figure 1. tRNA-mRNA in the P-site of the 70S ribosome.
(**a**) Superposition of the $tRNA_2^{Leu}$ anticodon-loop from the complex with the first P-site G•U mismatch on the $tRNA^{Phe}$ anticodon loop from the cognate complex (PDB code 3I8H) demonstrates that the geometry of the codon-anticodon duplex with the G•U mismatch is nearly identical to the cognate one. (**b**) The tight ribosomal "grip" around the anticodon stem-loop of tRNA in the P-site; van der Waals surfaces of proximal tRNA and rRNA elements are shown. (**c, d**) The sugar-phosphate backbone of the P-site codon is tightly fixed in place by conserved nucleotides G926, U1498, C1402 and C1403 of 16S rRNA (contacts from 1 to 5).

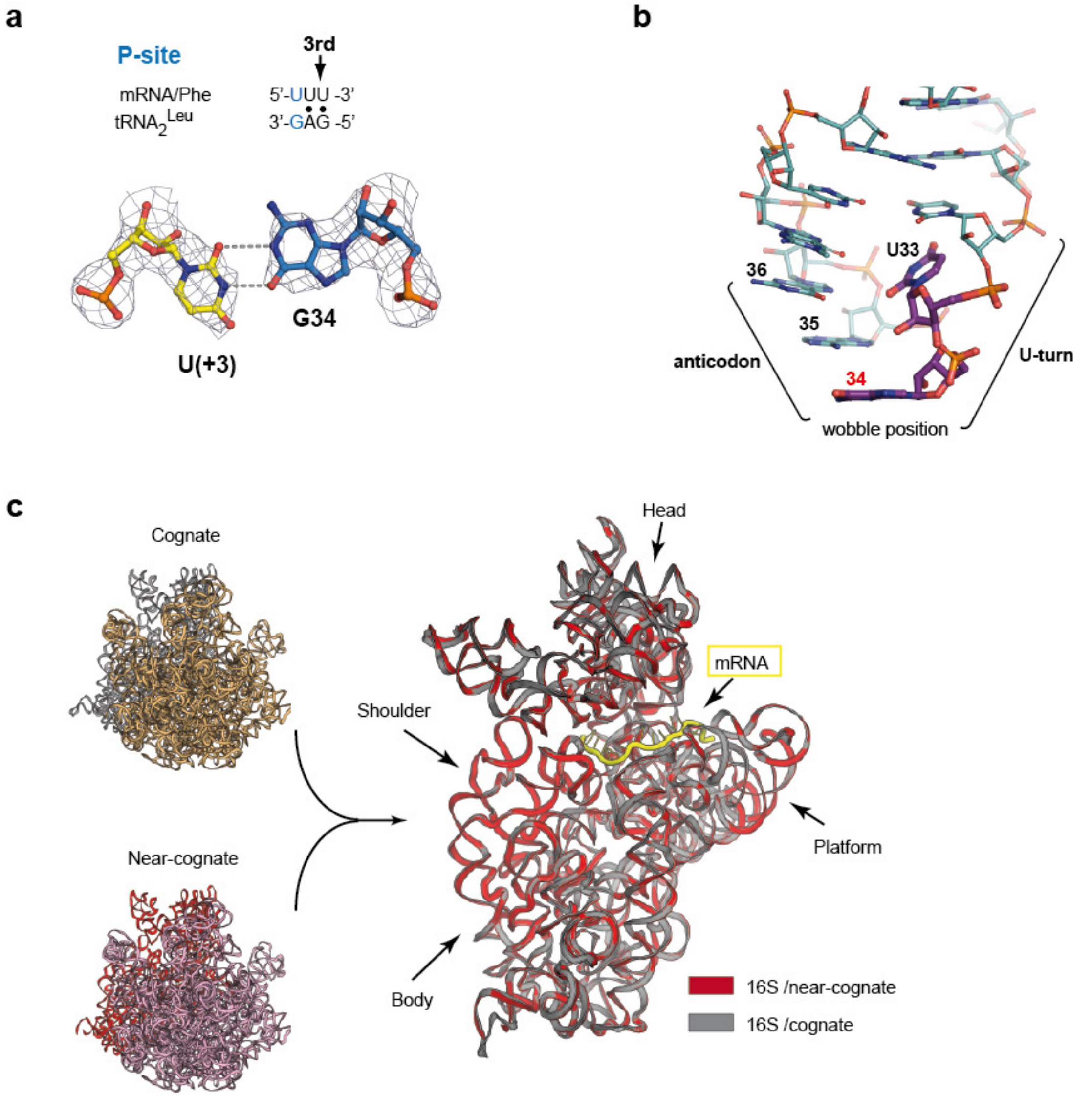

Supplementary figure 2.

(**a**) The G•U mismatch adopts canonical wobble geometry at the third position of the codon-anticodon duplex (arrow). Density maps are contoured at 1.6 – 1.8 σ level.

(**b**) Structure of the tRNA anticodon loop. The apical position of nucleotide 34 in the U-turn motif of $tRNA^{Phe}$; in a U-turn the phosphate backbone between the key uridine 33 and nucleotide 34 is turned 120 degrees from the standard helical conformation

(**c**) Near-cognate and cognate tRNAs induce identical rearrangements of the small ribosomal subunit. Superposition of 23S rRNA from the near-cognate structure with the first A•A mismatch in the codon-anticodon duplex in the decoding center and cognate model with $tRNA^{Tyr}$ (PDB codes 3UZ6 and 3UZ9) exemplifies identical conformations of 16S rRNA in the near-cognate and cognate states including the conformation of the shoulder domain. For clarity of the representation ribosomal proteins are not shown; the main domains of the small subunit and mRNA are indicated by arrows.

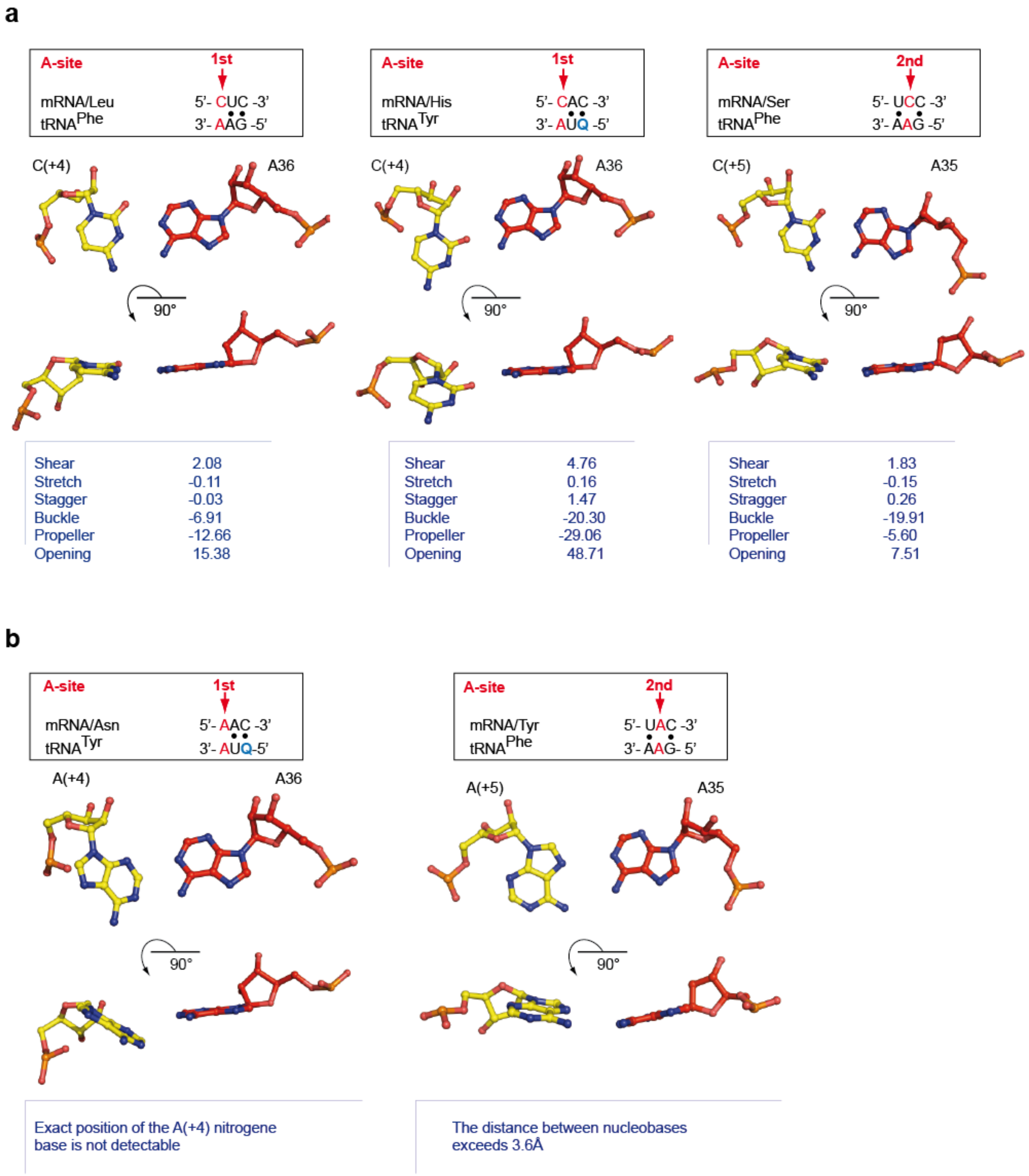

Supplementary figure 3. Absence of strong interactions in the C•A and A•A pairs at the first two positions of the codon-anticodon duplex bound in the 70S-decoding center. (**a**) Deviation of the C•A pair from the standard Watson-Crick geometry is specified by the intra-base-pair parameters presented below each panel. (**b**) Position of the codon adenosine could not be defined due to the lack of the corresponding electron density signal (left); the distance between nucleobases reflects very weak electrostatic interactions (right). In (a,b) the schemes of the codon-anticodon duplexes are indicated and arrows mark the described mispairs.

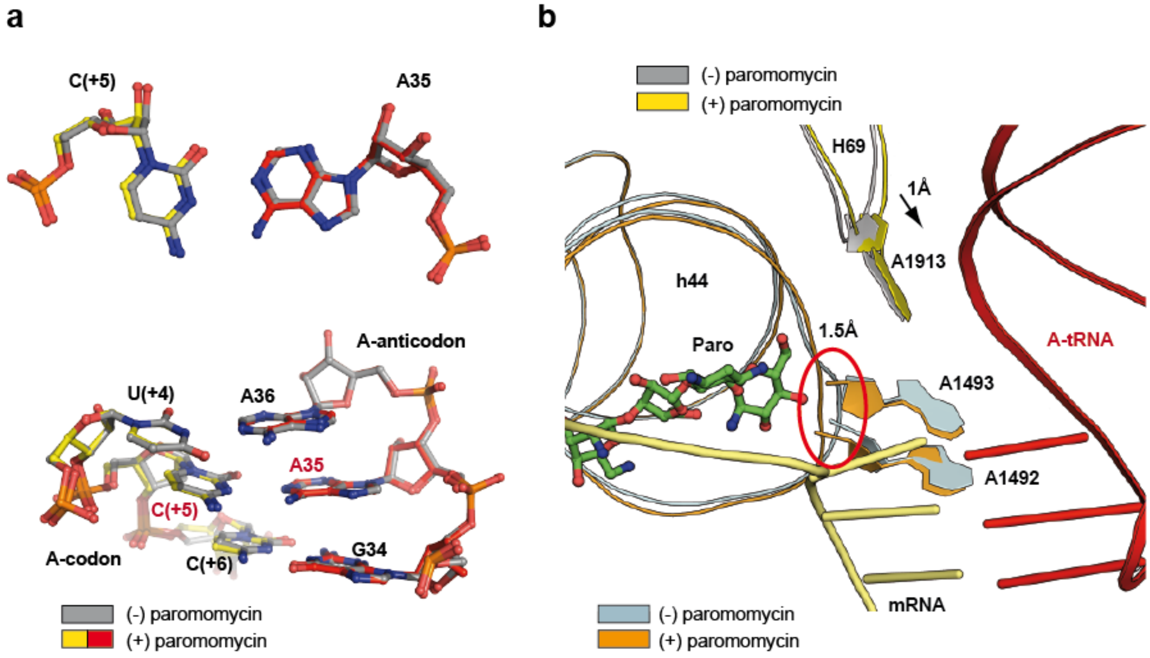

Supplementary figure 4. Aminoglycoside antibiotic paromomycin modulates the restrictive property of the 70S ribosomal decoding center. (**a**) Paromomycin does not affect the C•A mismatch geometry (top) in the codon-anticodon mini-helix (bottom); the $tRNA^{Phe}$ anticodon loop from the 70S structure with paromomycin and the C•A mismatch at the second codon-anticodon position was superimposed on the $tRNA^{Phe}$ anticodon loop from the analogous model obtained without antibiotic (Fig. 2c). (**b**) Paromomycin (Paro) binding leads to the 1.5 Å displacement of the A1493 phosphate group and rearrangement of the B2a bridge composed of h44 of 16S and H69 of 23S rRNA. The resultant shift of H69 towards incorrect tRNA can contribute to complementary stabilization of the latter on the ribosome. Superimposition was performed using 23S rRNA as reference.

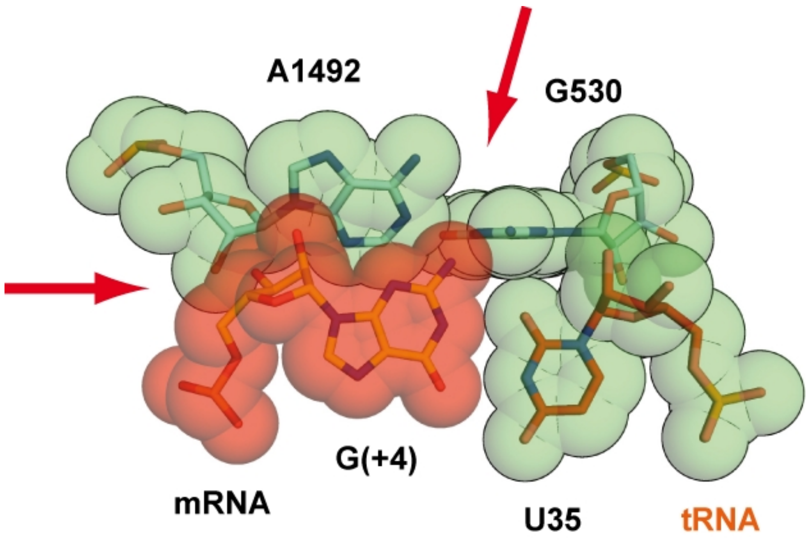

Supplementary figure 5. Steric clashes of a hypothetical wobble G•U pair in the second position of the codon-anticodon helix with A1492 and G530 of the decoding center of the 70S ribosome. Comparison of various mismatches in the codon-anticodon duplex reveals that the tRNA nucleotides are tightly fixed in place, therefore we presume to model wobble geometry by the shift of the G nucleotide in the A-codon.

Supplementary Table 1 Data collection and refinement statistics

	A-site:1st CA_1(Q)[1,*]	A-site:1st CA_2[2]	A-site:1st AA[3]	A-site:1st AA+paro[4]	A-site:2nd CA[5]
PDB ID	4WQ1	4WQR	4WR6	4WRA	4WRO
**Data collection**					
Space group	$P2_12_12_1$	$P2_12_12_1$	$P2_12_12_1$	$P2_12_12_1$	$P2_12_12_1$
Cell dimensions					
a, *b*, *c* (Å)	209.9, 448.7, 618.5	210.1, 448.8, 621.3	209.9, 450.3, 619.5	209.9, 450.9, 622.7	209.4, 447.7, 619.4
α, β, γ(°)	90.0, 90.0, 90.0	90.0, 90.0, 90.0	90.0, 90.0, 90.0	90.0, 90.0, 90.0	90.0, 90.0, 90.0
Resolution (Å)	300-3.1 (3.2-3.1)**	250-3.15 (3.25-3.15)	300-3.05 (3.15-3.05)	300-3.05 (3.15-3.05)	200-3.05 (3.15-3.05)
R_{meas} (%)	40.7 (391.9)	37.3 (479.8)	42.4 (377.4)	38.8 (440.6)	32.7 (462.9)
$I / \sigma I$	16.08 (1.32)	15.45 (1.19)	14.68(0.97)	9.57 (0.91)	14.16 (0.97)
CC1/2***	99.9 (45.8)	99.9(44.4)	99.9 (29.5)	99.8 (30.7)	99.9 (39.4)
Completeness (%)	99.9 (99.9)	99.9 (99.9)	99.9 (99.9)	99.9 (99.9)	99.9 (99.9)
Redundancy	79.7 (33.0)	83.5 (54.5)	76.6 (17.7)	30.9 (17.4)	63.8 (36.2)
**Refinement**					
Resolution (Å)	151.80-3.10	152.19-3.15	225.15-3.05	225.45-3.05	151.956-3.05
No. reflections	1043560	1000621	1100751	1107970	1091914
R_{work} / R_{free} (%)	18.82 / 23.46	18.92 / 23.12	20.05 / 24.91	19.48 / 24.89	19.33 / 23.07
No. atoms					
RNA	205549	205191	206210	206353	205183
Protein	91779	91870	90779	90584	91757
Ligands/water	2623	3509	2329	2671	3797
Average *B* factors					
RNA	102.36	113.77	102.07	100.83	114.36
Protein	113.82	119.32	110.39	103.48	118.44
Ligands/water	71.65	79.87	67.23	68.89	83.07
R.m.s. deviations					
Bond lengths (Å)	0.0101	0.011	0.0099	0.0094	0.011
Bond angles (°)	1.735	1.856	1.707	1.668	1.863

Supplementary Table 1 (continued) Data collection and refinement statistics

	A-site:2nd CA+paro[6]	A-site:2nd AA[7]	P-site:1st GU[8]	P-site:2nd GU[9]	P-site:2nd GC[10]
PDB ID	4WSD	4WT1	4WSM	4WU1	4WZD
**Data collection**					
Space group	$P2_12_12_1$	$P2_12_12_1$	$P2_12_12_1$	$P2_12_12_1$	$P2_12_12_1$
Cell dimensions					
a, *b*, *c* (Å)	209.4, 449.2, 621.2	209.9, 447.9, 621.5	209.1, 449.3, 618.1	210.0, 451.5, 616.7	210.7, 448.4, 616.3
α, β, γ(°)	90.0, 90.0, 90.0	90.0, 90.0, 90.0	90.0, 90.0, 90.0	90.0, 90.0, 90.0	90.0, 90.0, 90.0
Resolution (Å)	300-2.95 (3.05-2.95)	250-3.05 (3.15-3.05)	200-3.3 (3.4-3.3)	300-3.2 (3.3-3.2)	300-3.1 (3.2-3.1)
R_{meas} (%)	30.2 (426.6)	27.9 (552.7)	54.1 (504.9)	37.1 (386.2)	22.4 (307.2)
I / σI	18.67 (1.13)	24.41 (1.08)	9.1 (1.51)	14.09 (1.11)	10.31 (1.03)
CC1/2***	100 (44.4)	100 (58.2)	99.8 (48.3)	99.9 (40.8)	99.9 (37.0)
Completeness (%)	99.9 (99.8)	99.8 (99.4)	99.9 (100)	100 (100)	99.8 (99.6)
Redundancy	74.5 (29.0)	155.8 (67.5)	58.0 (52.9)	75.7 (22.3)	14.0 (13.7)
**Refinement**					
Resolution (Å)	255.475-2.95	188.03-3.05	153.31-3.30	198.79-3.20	153.54-3.10
No. reflections	1213202	1095536	866987	953058	1041073
R_{work} / R_{free}	19.33 / 23.49	19.94 / 23.86	18.53 / 23.13	18.77 / 23.82	19.31 / 22.75
No. atoms					
RNA	205286	205228	206640	202756	202845
Protein	91634	91684	90183	93268	94004
Ligands/water	3332	3039	2607	3656	2857
Average *B* factors					
RNA	102.97	127.57	115.55	123.82	111.39
Protein	108.51	132.32	118.51	132.76	123.46
Ligands/water	71.52	92.23	82.78	93.28	81.24
R.m.s. deviations					
Bond lengths (Å)	0.011	0.01	0.0089	0.0104	0.0113
Bond angles (°)	1.920	1.737	1.538	1.673	1.823

Supplementary Table 1 (continued) Data collection and refinement statistics

	A-site:Control[11]
PDB ID	4WZO
**Data collection**	
Space group	$P2_12_12_1$
Cell dimensions	
a, *b*, *c* (Å)	210.2, 448.8, 621.0
α, β, γ(°)	90.0, 90.0, 90.0
Resolution (Å)	300-3.3 (3.4-3.3)
R_{meas} (%)	46.5 (379.5)
$I / \sigma I$	9.41 (0.99)
CC1/2***	99.8 (35.3)
Completeness (%)	99.9 (99.9)
Redundancy	41.2 (19.9)
**Refinement**	
Resolution (Å)	153.41-3.30
No. reflections	871006
R_{work} / R_{free}	19.09 / 25.78
No. atoms	
RNA	202590
Protein	90503
Ligands/water	2827
Average *B* factors	
RNA	114.56
Protein	117.80
Ligands/water	79.72
R.m.s. deviations	
Bond lengths (Å)	0.0106
Bond angles (°)	1.773

* Q - queuosine modification on $tRNA^{Tyr}$

**Values in parentheses are for highest-resolution shell

***Half-dataset correlation coefficient[1]

Number of crystals used for data collection: [1] 8, [2] 6, [3] 14, [4] 7, [5] 9, [6] 8, [7] 17, [8] 7, [9] 10, [10] 7, [11] 5

Supplementary Movie

Conformational changes accompanying formation of the decoding center on the 70S ribosome.

The movie introduces tRNA binding sites on the 70S ribosome and highlights specific role of helix 69 from the large ribosomal subunit in formation of the A-tRNA binding site. The rearrangements of the ribosome demonstrate step-wise formation of the decoding center that occurs upon binding of any tRNA ligand to the 70S ribosome. The movie also represents slight movement of the shoulder domain that constitutes an important part in formation of the decoding center ‘mold’. The movie was created using PyMol.

Supplementary reference

1. Karplus, P.A. & Diederichs, K. Linking crystallographic model and data quality. *Science* **336**, 1030-3 (2012).
